# The Association Between Previous Suicide Attempts and Risk Level According to the Static‐99R in Men Who Have Sexually Offended

**DOI:** 10.1002/cbm.70012

**Published:** 2025-10-03

**Authors:** Jordyn Monaghan, Krystal Lowe, Michael C. Seto, Marc W. Patry, Skye Stephens

**Affiliations:** ^1^ Department of Psychology Saint Mary's University Halifax Canada; ^2^ The Royal's Institute for Mental Health Research Ottawa Canada

**Keywords:** antisociality, atypical sexual interest, previous suicide attempt, risk level, sexual offending, Static‐99R

## Abstract

**Background:**

Suicide is a leading cause of death within correctional institutions, with men convicted of sexual offences over‐represented among those who have attempted suicide. Despite an increased risk for suicidality, limited research has examined how past suicide attempts are associated with sexual offending and specific risk domains on assessment tools.

**Aim:**

In consideration of shared risk factors for suicidality and sexual offending, the present study examined the association between previous suicide attempts and sexual offending, particularly in terms of risk domains.

**Methods:**

The study sample comprised 369 men who underwent a comprehensive psychosexual assessment at a large sexual behaviour clinic between 1995 and 2006. Suicide attempt history was coded from clinical files and the association with Static‐99R scores, recidivism, sexual interest in children, antisocial traits and diagnosis, and demographic variables was examined.

**Results:**

Of our participants, 12.5% self‐reported having previously attempted suicide. Although there was no significant association between Static‐99R scores and previous suicide attempts, the magnitude of the effect was the same as previous research. Previous suicide attempts were significantly associated with an individual's age, prior non‐sexual violence, and four or more previous sentencing dates.

**Conclusion:**

Given the limited research in this area, the findings are important for increasing the understanding of suicide risk among people who have sexually offended. Study results suggest the need for an increasing understanding of suicide risk and more targeted suicide assessment and management strategies for those with sexual convictions.

## 1 Introduction

Suicide is a leading cause of preventable death within correctional institutions, particularly among individuals who have sexually offended (Fazel et al. 2018; Jeglic et al. 2013; Marzano et al. 2016; Pritchard and King 2005; World Health Organization 2007). There are shared risk factors for suicidal ideation and sexual offending (e.g., depression, hopelessness and shame; Cortoni and Marshall 2001; Jeglic et al. 2011), and it would be expected that measures used to assess the risk of sexual recidivism might be closely related to suicide risk as well. If individuals with a history of suicide attempts show elevations in particular risk domains, it could inform suicide risk assessment and management strategies.

### 1.1 Suicide and Actuarial Risk Assessment

To our knowledge, one study examined the relationship between suicide and actuarial risk for sexual offending. In a sample of 3040 males who had sexually offended, Jeglic et al. (2013) found that 14% (*n* = 406) had a prior suicide attempt (most attempts were prior to incarceration). These individuals scored higher on actuarial risk assessment tools, which included the Static‐99R, one of the most widely used actuarial sexual recidivism risk assessment tools (Hanson and Morton‐Bourgon 2009). Although Jeglic et al. (2013) found an association between suicide attempts and actuarial risk, they did not examine if a suicide attempt was associated with sexual recidivism, which is relevant, as suicidality is a risk factor in structural professional judgement tools (e.g., Risk for Sexual Violence Protocol; Hart et al. 2003). Further, they did not explore underlying risk dimensions reflected on the Static‐99R, such as atypical sexuality (e.g., paraphilia or sexual self‐regulation problems) and antisociality (e.g., impulsivity, recklessness and antisocial attitudes and beliefs).

The first major risk domain, atypical sexuality, refers to sexual interests that are non‐normative, such as a sexual interest in prepubescent or pubescent children. We expect that atypical sexuality is associated with suicide and that risk factors for suicide attempts (e.g., hopelessness, childhood abuse, substance use disorder and impulse control issues; Nock et al. 2010; Oquendo et al. 2006) are elevated among those with a sexual interest in children (e.g., Cohen et al. 2020; Levenson and Grady 2019; McPhail and Stephens 2024; Stephens et al. 2023). Moreover, intense stigma is associated with sexual interest in children (Jahnke 2018), which could be linked to suicidality. Past suicide attempts are elevated among those who had victimised boys or both boys and girls (Jeglic et al. 2013), both of which are associated with a greater likelihood of pedophilia (e.g., Levenson et al. 2008).

The second major risk dimension, antisociality, is characterised by impulsivity, aggression and substance use, which is highly correlated with sexual recidivism, suicidal ideation and suicide attempts (Fisher and Hany 2023; Gvion and Apter 2011; Hanson and Morton‐Bourgon 2004). Additionally, antisociality is strongly related to an increased risk of violence directed at others or oneself (Douglas et al. 2008; Verona et al. 2001; Webb et al. 2012). Despite this overlap, to our knowledge, no prior research has explored the connection between antisociality and suicide in those with sexual offence convictions.

### 1.2 Present Study

This study investigated the association between suicide attempts and risk of sexual recidivism via two research questions: (1) What is the association between previous suicide attempts and Static‐99R risk scores, and (2) What is the association between previous suicide attempts and specific risk domains such as sexual interest in children and antisociality? In line with Jeglic et al. (2013), we expected a positive correlation between Static‐99R total scores and prior suicide attempts. Next, we anticipated a strong positive association between previous suicide attempts and sexual interest in children as well as antisociality. Exploratory analyses tested whether suicide attempts added incremental validity to the Static‐99R in the prediction of violent and sexual recidivism.

## 2 Method

### 2.1 Sample

The study sample comprised 369 adult males who attended a large outpatient sexual behaviour clinic in a Canadian city between 1995 and 2006. All clients underwent a comprehensive assessment that included a psychosexual assessment, with an unknown proportion proceeding to treatment at the clinic. Clients were referred to the clinic if they had a sexually motivated offence (inclusive of online offences); however, self‐referrals were also possible. All individuals in the study had a history of sexual offending.

This study uses data from a large research database of 5006 individuals assessed at the clinic who consented to the inclusion of their assessment results in the database. Adult males were included in the recidivism study from this larger archival database (*n* = 1117 criminal records were ordered with 844 returned; see Moss et al. 2022). After the completion of the recidivism study, additional information was coded from client charts on clinically relevant factors. Of the 844 with criminal record data, only 369 had electronic clinical charts that were available for coding, which is the sample available for analysis. On average, participants were 39.66 (SD = 13.12) years old at the assessment (see Table 1 for additional demographic variables). The present sample is part of a larger sample used in previous recidivism studies that addressed distinct research questions (Faitakis et al. 2023; Moss et al. 2022; Stephens et al. 2017b, 2018).

**TABLE 1:** Sample demographic information.

Variables	*n*	%
Education (*N* = 367)		
< High school	172	46.9
High school graduate	91	24.8
College/University+	104	28.3
Ethnicity (*N* = 364)		
White	286	78.6
Non‐White	78	21.4
Referral type (*N* = 328)		
Probation/Parole	211	64.3
Own lawyer	36	10.9
Legal aid	2	0.01
Institution	74	22.6
Self‐referral	5	1.5

### 2.2 Measures

#### 2.2.1 Past Suicide Attempts

Past suicide attempts (present or absent) were coded from clinical assessment reports and the coding of this variable was consistent with previous research (Jeglic et al. 2013). Coders only coded explicit mention of suicide attempts (not suicidal ideation or plans) as this is a more significant indicator of suicidality. Given the centrality of questions about suicide during assessment, it was assumed that no mention in the clinical file represented the absence of past suicide attempts (missing data for seven participants was coded as no).

#### 2.2.2 Static‐99R

The Static‐99R (Phenix et al. 2016) is an actuarial risk assessment tool for sexual recidivism comprised of 10 factors (e.g., age at release, stranger victim, prior sex offences). The Static‐99R is associated with other sexual risk assessment tools and has moderate predictive validity (e.g., Hanson and Morton‐Bourgon 2009; Helmus et al. 2022; Vargen et al. 2020). Total Static‐99R scores were calculated retrospectively using official file information and criminal records. One of the coders received certified training on the measure, and the other was trained by the study investigator.^1^

#### 2.2.3 Sexual Interest in Children

Three variables in the archival database were used for sexual interest in children. First, self‐reported sexual interest was canvassed immediately before (in some cases after) phallometric testing by the technician using a standardised protocol that has been in use in the clinic since 1995. Individuals rated their sexual interest to males and females in the following categories: prepubescent (both under 5 and 6–10), pubescent (11 and 12–14), post‐pubescent teens (15–16) and adults (17 or older) from no sexual interest (1) to maximum sexual interest (5). The maximum score for prepubescent or pubescent children was used in the study as a quasi‐continuous measure of sexual interest in children (see Blanchard et al. 2009 for further details).

Volumetric phallometric testing (see Blanchard et al. 2001 for detailed information) was another indicator of sexual interest in children in the study. Penile blood volume change was measured in response to photographs of models that differed based on sex (male or female) and sexual maturity (prepubescent, pubescent or adult). While viewing these photographs, audiotaped narratives describing sexual interactions with a person who matched the stimuli were presented via headphones. There were four trials per stimulus category (28 trials). The test took one to 2 hours to complete. The test was invalid if individuals responded more to the neutral category (control condition comprised of pictures of landscapes) or had a minimal sexual response (defined as 1 cc; Lykins et al. 2010). Phallometric data were reduced to seven category z‐scores, and a pedohebephilia index was computed (maximum *z*‐score for prepubescent or pubescent stimuli minus the maximum *z*‐score for adult stimuli, regardless of sex). The pedohebephilia index was continuous, with higher scores representing greater arousal to children, relative to adults. Research has provided extensive support for the validity of the phallometric procedures (e.g., Blanchard et al. 2001, 2009; Stephens, Cantor, et al. 2017a).

Lastly, the Screening Scale of Pedophilic Interest‐Revised (SSPI‐2) was scored retrospectively based on the information contained in the archival database. The SSPI‐2 is comprised of five items (present or absent): having a boy child victim, having multiple child victims, having any young child victims under age 12, having extrafamilial child victims and the use of child sexual exploitation material (Seto, Stephens, et al. 2017b). The SSPI‐2 ranges from a score of zero to five. The SSPI‐2 score is associated with other indicators of sexual interest in children (e.g., Seto, Stephens, et al. 2017b) and sexual recidivism (Faitakis et al. 2023; Seto et al. 2017a).

#### 2.2.4 Antisociality

Clinician reports were coded for the presence of antisocial personality disorder or traits of antisocial personality disorder based on the Diagnostic and Statistical Manual of Mental Disorders *(DSM)* that was in use at the time of the assessment (either *DSM‐IV* or *DSM‐IV‐TR* criteria; American Psychiatric Association 1994, 2000). Antisocial personality disorder was coded as present if the client met the criteria for the full disorder, whereas traits were only coded as present if the clinician indicated that there were traits or features of the disorder in their diagnostic write‐up.

#### 2.2.5 Recidivism

Recidivism was coded via national criminal records. All individuals were at risk in the community for at least part of the follow‐up period. We considered sexual recidivism (sexual contact and non‐contact, such as sexual assault, luring and child pornography) and violent recidivism (e.g., assault) if there was a charge and/or conviction that occurred after the assessment data. The start of the follow‐up period and follow‐up time were recorded in months. The end of the follow‐up period was the date the records were received unless the person had died, in which case, the date of death was the end of the follow‐up period. Time in secure custody was subtracted from the follow‐up period to account for the opportunity to reoffend.

### 2.3 Procedure and Analysis

Information on demographic variables, sexual interest in children indicators and sexual offending history was contained in an archival database maintained at the clinic by a single research technician (no ICC available for these variables). The last author and an upper‐year undergraduate student coded recidivism information and information from available clinical files for recidivism, suicidality and diagnostic variables. All ICC values for the recidivism and clinical coding variables ranged from 0.75 (violent reoffence convictions) to 0.99 (violent charge date). To address the research questions, a series of *t*‐tests, Chi‐squared, and Cox regression tests were conducted.

## 3 Results

Prior suicide attempts were present among 46 individuals (12.5%). The mean Static‐99R total score was 2.35 (SD = 2.55), and the average follow‐up time was 95.00 months (SD = 42.98). The sexual recidivism rate was 14.9% (*n* = 51), and violent recidivism (including sexual violence) was 22.2% (*n* = 76). The pedohebephilia index Z score average was 0.004 (SD = 0.85), with a slight positive score indicating greater arousal to children. The SSPI‐2 mean score for this sample was 1.93 (SD = 1.47), with higher scores indicating sexual interest in children on the five‐point scale. Participants had an average self‐reported sexual preference of 1.60 (SD = 1.25). For antisociality, 6.2% (*n* = 20) had an antisocial personality disorder diagnosis, and 20.5% (*n* = 66) had traits of antisociality or a diagnosis.

Those with a previous suicide attempt were younger at the time of the assessment (*M* = 35.80, SD = 10.3) compared with those without a history of an attempt (*M* = 40.20, SD = 13.39), *t* (365) = 2.12 and *p* = 0.035, with a small effect size, *d* = 0.34. There was no significant difference in the number of victims between those with a previous attempt (*M* = 3.07, SD = 2.74) compared with those without an attempt (*M* = 3.02, SD = 2.78), *t* (364) = −0.10 and *p* = 0.923, *d* = −0.02. Prior nonsexual violence was associated with previous suicide attempts and previous sentencing dates (see Table 2).

**TABLE 2:** Descriptive information.

	Previous attempt		No previous attempt		*X* ^2^	*p*	Cramer's *V*
	*n*	%	*n*	%			
Child victim under 15 (*N* = 366)					0.51	0.475	0.04
At least one child victim (*n* = 293)	37	84.1	256	79.5			
No child victim (*n* = 73)	7	15.9	66	20.5			
Child victim gender (*N* = 293)					1.29	0.524	0.07
Boy under 15 (*n* = 50)	5	13.5	45	17.6			
Girl under 15 (*n* = 197)	24	64.9	173	67.6			
Boy and girl under 15 (*n* = 46)	8	21.6	38	14.8			
Prior nonsexual violence (*N* = 361)					7.63	0.006	0.15
Prior (*n* = 68)	15	34.1	53	16.7			
No prior (*n* = 293)	29	65.9	264	83.3			
Prior sexual violence (*N* = 362)					0.80	0.370	0.05
Prior (*n* = 95)	14	31.8	81	25.5			
No prior (*n* = 267)	30	68.2	237	74.5			
# Sentencing dates (*N* = 361)					8.78	0.003	0.16
< 4 sentencing dates (*n* = 302)	30	68.2	272	85.8			
4+ sentencing dates (*n* = 59)	14	31.8	45	14.2			

### 3.1 Suicide Attempts and Sexual Recidivism Risk

There was no statistically significant difference in Static‐99R total scores between those with (*M* = 2.88, SD = 2.17) and without previous suicide attempts (*M* = 2.28, SD = 2.59), *t* (320) = −1.41 and *p* = 0.159, with a small effect size, *d* = −0.24. Further, there was no statistically significant difference in sexual interest in children (see Table 3) or antisociality (see Table 4) between those with previous suicide attempts and those without. Because of a violation of normality, Mann–Whitney *U* test was conducted for self‐reported sexual preference, which found no significant difference between those with (*Med* = 1, IQR = 2) and without previous suicide attempts (*Med* = 1, IQR = 0), *U* = 6192.50 and *p* = 0.057, with a negligible effect size *r* = −0.10.

**TABLE 3:** Atypical sexual interests and previous suicide attempts.

	Previous attempt		No previous attempt		*t*	*p*	Cohen's *d*
	*M* (*n*)	SD	*M (n)*	SD			
Pedohebephilia index (*N* = 356)	−0.10 (43)	1.02	0.02 (313)	0.84	0.87	0.386	0.14
SSPI‐2 total score (*N* = 366)	2.14 (44)	1.42	1.91 (322)	1.48	−0.97	0.333	−0.16

**TABLE 4:** Antisociality and previous suicide attempts.

	Previous attempt		No previous attempt		*X* ^2^	*p*	*V*	OR [95% CI]
	*n*	%	*n*	%				
ASPD diagnosis (*N* = 322)					0.31	0.478	0.03	1.44 [0.40, 5.17]
Diagnosis (*n* = 20)	3	8.3	17	5.9				
No diagnosis (*n* = 302)	33	91.7	269	94.1				
Antisocial traits (*N* = 322)					0.07	0.786	0.02	1.12 [0.49, 2.59]
Traits (*n* = 66)	8	22.2	58	20.3				
No traits (*n* = 256)	28	77.8	228	79.7				

*Note:* Antisocial traits include those with traits or an official diagnosis. Fisher's exact test was used for the ASPD analysis because of the small cell size.

Abbreviation: ASPD, Antisocial Personality Disorder diagnosis.

### 3.2 Suicide Attempts and Recidivism

Prior suicide attempts were not associated with sexual or violent recidivism (see Table 5). Incremental validity was not examined due to the absence of significant bivariate associations.

**TABLE 5:** Recidivism and previous suicide attempts (*n* = 338).

	B (SE)	Wald	HR [95% CI]
Sexual recidivism	0.42 (0.37)	1.29	1.52 [0.74, 3.12]
Violent recidivism	−0.21 (0.37)	0.33	0.81 [0.39, 1.68]

## 4 Discussion

The present study explored the association between previous suicide attempts, actuarial risk and recidivism. In our sample, 12.5% of individuals had a history of suicide attempts, which is similar to past research with a large American sample of men who had sexually offended (14% in Jeglic et al. 2013); however, the rate of suicide attempts was lower in our sample than data reported by clients who had sexually offended (19%; Katsman and Jeglic 2020) and other countries (e.g., Australia, 23%; Gullotta et al. 2021). Overall, past rates have ranged from 12.5% to 23%, which is substantially higher than the public (3.1%; Public Health Agency of Canada 2023) and among those with a history of non‐sexual non‐violent offending (15% compared to 23% of those with sexual offences in the same sample; Gullotta et al. 2021). These results suggest that suicide is of particular concern among those with a sexual offence history.

### 4.1 Implications of Findings on Risk and Risk Factors

The effect size between past suicide attempts and actuarial risk for sexual recidivism in the present study is consistent with Jeglic et al. (2013) in that those with a past suicide attempt scored 0.5 points higher on the Static‐99R. We extended Jeglic et al. (2013) and found that past suicide attempts were not significantly associated with sexual or violent recidivism and sexual interest in children. Although antisociality was not significantly associated with suicidality, some of the criminal history indicators suggestive of antisociality were related to suicide (prior non‐sexual violence or prior sentencing dates). The results for past involvement with the criminal justice system are consistent with past findings and suggest that these factors may be more important indicators of suicidality in those who sexually offend (Gullotta et al. 2021; Jeglic et al. 2013). Similar to previous research (Cohen et al. 2020), our findings indicated that an individual's age is associated with having a previous suicide attempt. Cohen et al. (2020) found that suicide risk is higher in young people due to increased impulsive aggression and substance use compared to older individuals who have more time to adjust to their atypical sexuality and develop strong coping skills.

The implications of our findings of an association between past suicide attempts and criminal history variables (past sentencing dates and non‐sexual violence) is worth highlighting as it is possible that these variables signify issues with impulsivity that could have driven the findings related to suicidality. Overall, empirical evidence suggests that impulsivity contributes to harmful behaviour (Cohen et al. 2020; Martin et al. 2019) and both self and other‐directed violence (O'Donnell et al. 2015). Although not the focus of this study, the finding that nonsexual violent offending behaviour and past involvement with the criminal justice system is related to past suicide attempts highlights the need to assess both internalising and externalising behaviours among justice‐involved individuals who may be at risk for suicide. These findings may have implications for the detection and prevention of suicide in this population because they suggest that past criminal history may be important to pay attention to regarding suicide risk.

Overall, our findings point to suicidality as an important and discrete clinical screening concern at intake, especially for individuals who have prior criminal justice experience. Suicidality is a basic concern for all clinicians working in correctional settings, and yet research has not identified clear predictors of suicide attempt lethality (e.g., Labrecque and Patry 2018; Magaletta et al. 2008), although violent offending may be indicative of heightened risk. Chronic suicide risk may be explained by a higher prevalence of mood disorders or stigma in men with sexual interest in children (e.g., Fox et al. 2022; Jahnke 2018) and/or concern about the potential discovery of the sexual offences and the related stigma (Cohen et al. 2020). The acute risk of suicidality comes in the period between disclosure or reporting and prosecution, and might be explained by the shame, embarrassment and social impact of being identified as someone who has sexually offended against children (e.g., social rejection or loss of employment; Cohen et al. 2020; Katsman and Jeglic 2020; Key et al. 2021). Clinicians need to assess both the chronic and acute risk factors for suicide and respond appropriately, ensuring that interventions are tailored to meet the complexities of suicidality in this population.

### 4.2 Limitations

There are notable limitations with our past suicide attempt variable, which was derived from archival clinical records. A more robust measure of suicidality and information on when it occurred in relation to justice system involvement would have complemented our analyses. There is also the issue of a selection bias, given that those who have died by suicide would not be included in the sample (notably, the criminal records suggested that 11.2% or 11 individuals in the sample died during the follow‐up period, though we have no further details about the nature of death). Further, there were only 369 individuals with clinical records to code from, so we were unable to generate a past suicide attempt rate among everyone seen at the clinic.

There were also limitations with other variables in the present study. The measures of atypical sexuality and antisociality were limited to sexual interest in children (excluding other paraphilic interests and hypersexuality) and a diagnosis of antisocial personality disorder (categorical variable that does not capture variation in the strength or prominence of specific traits). A continuous measure of impulsivity, for example, may have revealed the expected association between this trait and the risk of suicidality.

### 4.3 Future Research Directions

Future research should provide an extended examination of risk dimensions and suicidality in individuals who sexually offend, inclusive of well‐known risk factors for suicidality in other populations, including different measures of impulsivity. Optimally, this research would follow individuals prospectively to better understand the temporal sequencing of risk factors and suicidality. Relatedly, there is a need to examine whether established methods of suicide intervention and crisis supports are effective with those who sexually offend.

## Conflicts of Interest

The authors declare no conflicts of interest.

## Data Availability

Data are unavailable in order to uphold the informed consent statements that data would not be publicly available.

## Endnotes

^1^
The last author provided the training to the research assistant, which included a review of the manual, assigning practice cases for scoring and frequent check in and discussions regarding scoring. Raters were instructed to reference the manual when coding the Static‐99R and to have this physically in front of them as they scored the measure. A detailed log was kept that identified any scoring issues that were encountered during the project. There was also the opportunity to consult with the third author of the paper who was overseeing the project if any issues arose, inclusive of issues with scoring.
